# Sudden phototactic reversal induced by hypochlorous acid exposure in the bioluminescent ostracod crustacean, *Vargula hilgendorfii.*

**DOI:** 10.17912/micropub.biology.001410

**Published:** 2024-12-17

**Authors:** Kouki Sugitani, Reika Ohnishi, Daigo Inaoka, Hiroki Ono, Noritaka Hirohashi

**Affiliations:** 1 Shimane University, Matsue, Shimane, Japan

## Abstract

The reversal of phototaxis has been observed in a wide range of animal species. However, environmental chemicals that can cause a quick reversal of phototaxis have rarely been reported. Here we identified hypochlorous acid (HClO) as an inducer of phototactic reversal in *Vargula hilgendorfii* , also known as sea fireflies. This species shows innate photophobic swimming behavior away from light sources and 0.1% HClO triggers rapid positive phototactic responses, that is reversible upon washout. Phototactic assays using monochromatic light irradiation revealed the highest photophobic responses at 460 nm, while the HClO-induced photophilic responses were observed widely, even in UV light.

**Figure 1. Phototactic reversal by HClO in V. hilgendorfii:**
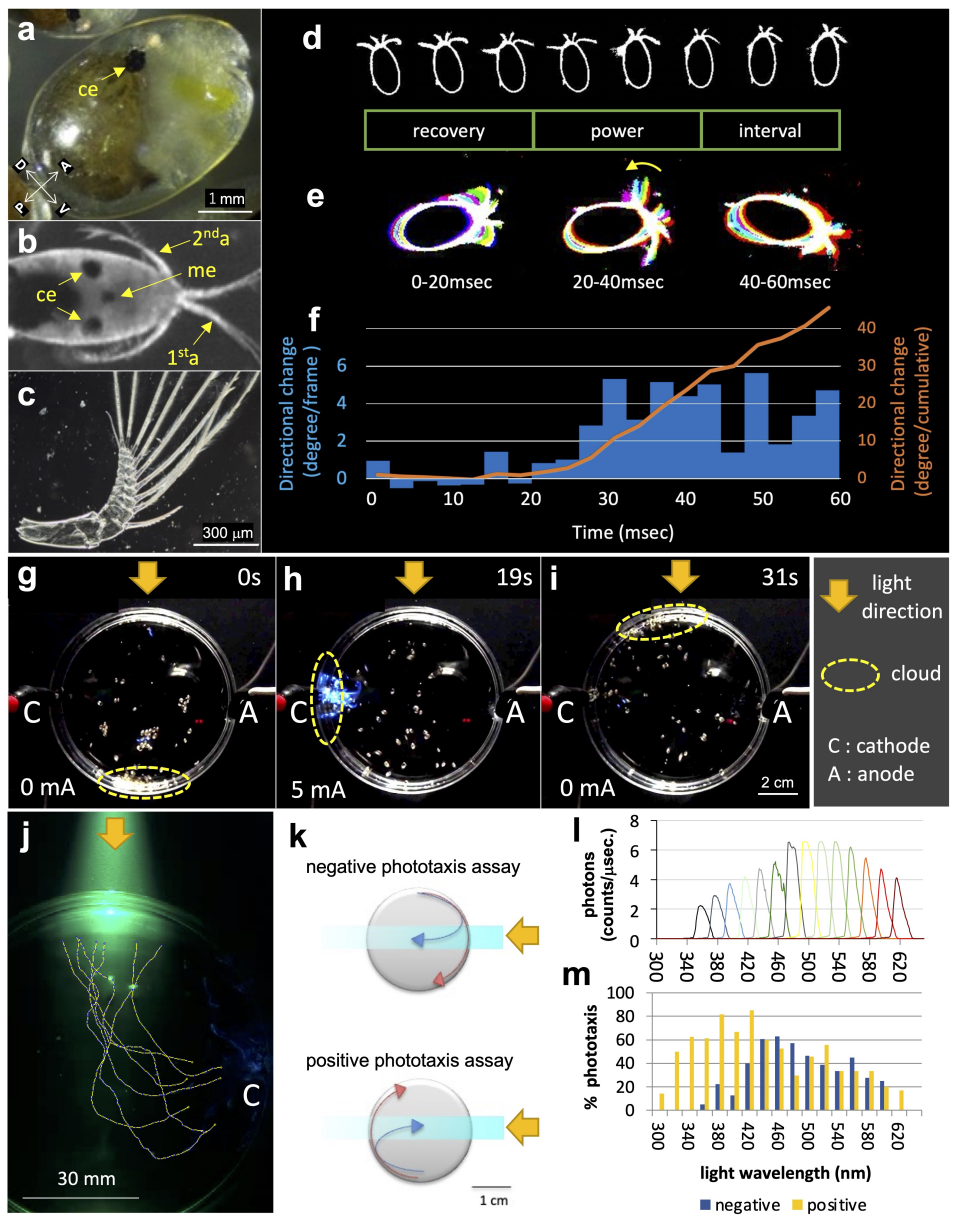
**a:** A side view of *V. hilgendorfii* showing the compound eye (ce) is depicted. Body axes with dorsoventral (D, V) and anterior-posterior (A, P) orientations are also shown. **b:** A dorsal view displaying a pair of compound eyes (ce), a median eye (me), and the 1st and 2nd appendages (1 ^st^ a, 2 ^nd^ a). **c:** Isolated 2nd appendage. **d-f:** Motion analysis of the 2nd appendages during a directional change in a free-swimming condition. Time-lapse frames ( **d,** *top* ) can identify recovery, power, and interval strokes ( **d,** *bottom* ) in a series of strokes. **e:** Superimposed images of the strokes exhibit asymmetrical movements during the power stroking episode ( *curved arrow* ). **f:** The frame-by-frame analysis showed the onset of reorientation during power strokes, after which the changing rates of direction remained consistent. **g-i:** Electric current-induced phototactic reversal. The swarming cloud of *V. hilgendorfii* appeared in a distal position from the light source ( **g** , photophobic), which was interfered with weak current (5mA, 5sec), resulting in accumulation at the cathode ( **h** , electrotaxis), followed by positive phototaxis ( **i** ). **j:** Shown are the representative swimming trajectories of seven individuals that were initially located near the cathode (C). Immediately after entering the light path, animals turned their swimming directions toward the source of light. **k:** Diagrams of phototaxis assays showing an 8-mm light path that penetrates a 30-mm petri-dish (see Methods for more detail). **l:** Spectra of the monochromatic irradiation used in the experiments to determine optical specificity. **m:** The optical specificity of phototactic responses in individuals treated with or without 0.1% hypochlorous acid.

## Description

Phototaxis is a fundamental innate behavior that is evolutionarily conserved in a wide range of organisms (Fraenkel & Gunn, 1961; Jékely, 2009) . The reversal of phototactic responses, i.e., from positive to negative or from negative to positive, is also a ubiquitous phenomenon in nature (Ranade, 1957; Minot, 1988; Mészáros et al., 2024). For example, male and female gametes of green algae exhibit positive phototaxis, which brings the gametes of opposite sex together at the water surface and facilitates their encounters (Cox, 1983; Togashi, et al., 1999) . Immediately after becoming zygotes, they swim down to the bottom of the water via negative phototaxis (Bliding, 1957) . There is a wealth of information about internal signals that are involved in phototactic reversal (Schmidt, 1976; Wakabayashi et al., 2011; Shiratori et al., 2017) . In contrast, there are only a few known external stimuli except for altered light intensities that can trigger phototactic reversal (Wodsedalek, 1911; Allee et al., 1918; Waterhouse, 1953; Shimizu et al., 1978; Hirschberg et al, 1980). We coincidentally found that the ostracod crustacean, *V. hilgendorfii* ( Fig. 1 a-c), showed a rapid change in phototactic behaviors — from negative to positive — upon applying a brief electric current ( Fig. 1 g-i, 5mA, 5 s, Supplemental data in figshare, Video3). This species shows innate photophobic swimming behavior away from light sources (Supplemental data in figshare, Video2). This reversed response was found to occur due to HClO being generated at the cathode rather than animals perceiving an electric shock. More specifically, although they showed positive electrotaxis and thereafter discharged bioluminescence around the cathode ( Fig. 1 h), these reactions were found unrelated to phototactic reversal. *V. hilgendorfii* propels itself using a pair of second appendages and changes swimming direction by breaking its symmetrical strokes ( Fig. 1 d-f, Supplemental data in figshare, Video1). After stopping the electric current, individuals near the cathode swam away in random directions. They were then reorientated toward a light source (positive phototaxis) as they entered the optical field ( Fig. 1 j, Supplemental data in figshare, Video5), suggesting that their positive phototactic response is based on the direction of light, rather than the intensity of light. To gain insight into photoreceptor characteristics, we investigated the spectral specificity of phototaxis using a two-way assay chamber that can detect either negative or positive phototaxis. ( Fig. 1 k-l). In conditions where animals exhibit negative phototaxis (Mock-treated), they showed a maximum negative response to light with a wavelength of around 460 nm. In contrast, in HClO-treated counterparts, the maximum positive phototactic responses were observed in a wavelength range between 380 and 420 nm. More importantly, positive phototaxis was observed in a wider wavelength range, including ultraviolet (UV) light ( Fig. 1 m). Behavioural avoidance from harmful UV irradiation is a ubiquitous and crucial phenomenon in animals. A flavoprotein called cryptochrome has been identified as a non-opsin blue/UV-A light photoreceptor in *Drosophila melanogaster* (Baik et al., 2017) . The planktonic ostracod, *Daphnia magna* , also possesses cryptochrome and exhibits positive phototaxis toward blue light (Itoh et al., 2010; Nitta et al., 2019) . Further studies should direct molecular mechanisms involving UV-sensitive photo-responses in *V. hilgendorfii* .

Since the effect of HClO was reversible, with the organisms returning to negative phototaxis after being replaceed in fresh seawater, and HClO covalently modulates primary amines such as dopamine (Palladino et al., 2020) , we speculate that this could potentially block specific synaptic connections in neuronal circuits. It is important for this species to detect not only light intensity but also light direction because they emit bioluminescence as the mating signal in the dark (Rivers et al., 2008) . Therefore, switching from negative to positive phototaxis may be a physiologically relevant phenomenon in this nocturnal species. It is known that HClO can be generated intrinsically (Liu et al., 2023) , which causes neurogenerative disorders in humans through chemical modification of dopamine (Jeitner et al., 2016) . The diel cycle of positive-negative phototaxis in marbled crayfish is controlled by periodical changes in the dopamine/serotonin ratio in the brain under the control of circadian rhythm (Shiratori et al., 2017) . Our current study may provide a novel system that can help us understand the kinematic, neuronal and molecular mechanisms underlying phototaxis and its reversal reaction.

## Methods

V. hilgendorfii were collected in Oki Island (Doh-go), Shimane, Japan using baited traps after sunset and kept in aquaria. In experiments for current-induced phototactic reversal, approximately 30 animals were placed in a 90-mm plastic plate filled with 10 mL of filtered seawater, and electrically shocked at 5mA for 5-10 sec using platinum electrodes. To investigate the optical specificity of phototaxis, we set up a spectral light irradiation system (Xenon short-arc lamp ballast with a grating instrument, Wacom Research and Development). The irradiation spectra (resolving power) and photon counts (light intensity) emitted through a monochromator were monitored using a spectrometer (USB4000, Ocean Optics) and OPwave+ (Ocean Photonics). Negative and positive phototaxis were tested using a 30-mm culture dish with an 8-mm light path (Fig1k). In the photo-positive assay, animals that made directional changes toward and ascended a light path were considered positive. Conversely, animals that crossed a light path were considered negative. Similarly, in the photo-negative assay, animals that turned away from a light path were considered to have negative phototactic responses. At least 20 individuals were examined for each trial at every 20 nm wavelength between 300 nm and 620 nm, for each treatment (with or without HClO). Experiments were duplicated with different batches and all data were integrated into a graph (Fig 1m). &nbsp; Data Availability Videos are available at https://figshare.com/articles/media/Video1_Free_swim_sea_fireflies_mov/27626298?file=50288436
